# Measurement of mean subcutaneous fat thickness: eight standardised ultrasound sites compared to 216 randomly selected sites

**DOI:** 10.1038/s41598-018-34213-0

**Published:** 2018-11-02

**Authors:** Paul Störchle, Wolfram Müller, Marietta Sengeis, Sonja Lackner, Sandra Holasek, Alfred Fürhapter-Rieger

**Affiliations:** 1 Medical University of Graz, Institute of Biophysics, Graz, Austria; 2 Medical University of Graz, Institute of Pathophysiology and Immunology, Graz, Austria

## Abstract

Ultrasound (US) provides the most accurate technique for thickness measurements of subcutaneous adipose tissue (SAT) layers. This US method was recently standardised using eight sites to capture SAT patterning and allows distinguishing between fat and embedded fibrous structures. These eight sites chosen for fat patterning studies do not represent the mean SAT thickness measured all over the body that is necessary for determining subcutaneous fat mass. This was obtained by SAT measurements at 216 sites distributed randomly all over the body. Ten participants with BMI below 28.5kgm^−2^ and SAT means (from eight sites) ranging from 3 mm to 10 mm were selected. The means from eight sites overestimated the means obtained from 216 sites (i.e. 2160 US measurements in the ten participants); the calibration factor of 0.65 corrects this; standard deviation (SD) was 0.05, i.e. 8%. The SD of the calibration factor transforms linearly when estimating the error range of the whole body’s SAT volume (body surface area times the calibrated mean SAT thickness). The SAT masses ranged from 3.2 to 12.4 kg in this group. The standard deviations resulting from solely the calibration factor uncertainty were ±0.3 and ±1.0 kg, respectively. For these examples, the SAT percentages were 4.9(±0.4)% and 13.3(±1.0)%.

## Introduction

A balanced body composition is an essential determinant of both health and physical performance. Non-communicable chronic diseases that are associated to inactivity and body composition disturbances are among the major public health problems of the 21^st^ century^1,2^. According to the World Health Organization (WHO), the worldwide prevalence of obesity nearly doubled between 1980 and 2014. Meanwhile more than 1.9 billion adults are overweight, and more than half a billion of them are obese^2^. Overweight and obesity were estimated to account for 3.4 million deaths per year and 93.6 million disability-adjusted life years in 2010^2,3^.

On the other hand, malnutrition and eating disorders can lead to underweight and body composition disturbances that are likely to result in severe diseases like anorexia nervosa, which is associated with alarming mental and physical implications and a high mortality rate^4–7^.

Better protection of health and the development of improved diagnostic criteria and intervention control depend on the availability of accurate and reliable methods for assessing body composition. Ultrasound has been established as an accurate and reliable method for measuring subcutaneous adipose tissue (SAT) thicknesses when used in standardised way.

## Body Composition Assessment Techniques

Over the past decades, many measurement techniques and equations have been developed for body composition assessment; among them are reference, laboratory, and field methods^8–10^. The body composition components can be analysed on the molecular or on the anatomical level.

The most accurate method for determining body fat on the molecular level is the 4-component model^8,11,12^ that measures the hydration status (D_2_O-diliution method), density (underwater weighing or plethysmography), bone mineral density (double X-ray absorptiometry, DXA), and determines the total body fat content (TBF) this way. Other methods like bio-impedance (BIA), or calculations based on skinfolds or near infrared spectroscopy, are associated with major measurement errors and therefore of limited value^8,11,13^. Also the X-ray based DXA method for fat measurement is far from being the gold standard, particularly in slim persons where severe measurement errors can occur; even “negative” amounts of fat on the trunk have been “measured”^8^. Manufacturers use different simplifying models and calculation algorithms which are necessary because two X-ray energies are not capable of distinguishing between three sorts of tissue (bone, muscle, and fat). This results in different “measurement values” depending on the machine used and also on the individual deviations from the type of “standard person” mapped in the algorithm. There is also a difference in pencil versus fan-beam accuracy, and according to Aragon *et al*.^11^, DXA may be unreliable for longitudinal studies of subjects who undergo major changes in glycogen or hydration status between measurements, when compared to the 4 component model^8,11^.

On the anatomical level, cadaver dissections, skinfold thickness measurements, or medical imaging techniques like MRI, CT, and US are in use. It has been shown recently that US provides the highest measurement accuracy for thickness measurement of SAT^13–17^ because image resolution can be as high as 0.1 mm (18 MHz probe) when using state-of-the art US equipment, whereas pixel size in MRI used for total body scans today is typically between 1.3 and 2.0 mm only^8^. However, there are many inherent problems involved in all measurement techniques and in the assumptions they make^8–11,13,18^. Skinfold methods for measuring SAT thickness are of limited value because fat is highly compressible, and skin thickness, which is included in skinfold measurements, varies from site to site and among individuals^8,13,15^. Many methods that are widely used in field studies are not standardised sufficiently. When the body composition status is assessed with multiple technologies, often results vary substantially because accuracy, reliability, or both are far from what should be expected from a good measurement technique^8,13^. Such results are confusing and not helpful for diagnosis or treatment control.

Particularly high demands on accuracy and reliability are necessary when investigating athletes, where body composition is a major performance determinant.

Inaccurate measurements of body composition and its changes would be confusing and misleading: most athletes have very low body fat and therefore very high accuracy and reliability is necessary for detecting the small changes of relevance for performance optimisation, and also for monitoring the health status of the athletes in order to prevent severe diseases like anorexia nervosa or other medical (and performance) problems associated with eating disorders^19–21^. Most recently, the best techniques for measuring body composition have been summarised in *Best Practice Protocols for Physique Assessment in Sport*^13^. Application of poor methods in terms of accuracy and reliability or both can be misleading, particularly in competitive sports, and also in other groups where body composition is a crucial health factor like in anorexia nervosa patients (this wide-spread severe disease is among the major medical problem in both female and male athletes)^8,15,19^.

## Ultrasound Measurement of Subcutaneous Adipose Tissue (SAT)

Adipose tissue is mainly stored in the subcutaneous region of the body, but significant amounts can also be found near organs (visceral adipose tissue, VAT), in the bone marrows, and within tissues, e.g. in muscle^9,22^. The amount of SAT ranges from only a few kilograms up to 50% of body weight and even more^17^. Adipose tissue consists of adipocytes and embedded collagen and elastin fibres which support the tissue^23^.

US was used for SAT thickness measurements in 1965 and 1966 already^24,25^. Bellisari *et al*. found that inter- and intra-observer errors were less than 0.15 mm at all of their investigated sites, except for triceps where they found 0.6 mm^26^. US has also been applied for measuring visceral fat. Koda *et al*.^27^ compared MRI measurements to US measurements of subcutaneous and visceral fat and they already found that US was highly accurate and reliable. 2016 a standardised US technique for measuring SAT has been presented^16,17^.

US is the only imaging method capable of measuring both the thickness of the fat layer with and without the embedded structures^14–16,28^. The applicability of this novel approach in groups with overweight and obesity has been shown by Störchle *et al*.^17^. Eight sites are used to represent the trunk (three), the arms (two), and the legs (three). US images of all sites show a simple structure: skin, SAT, muscle fascia. The sites were selected such that the thickness of the layer does not change appreciably in the vicinity of the site; this increases reliability. Site marking is easy and can be learned with high precision within short time (one hour training is sufficient). All distances necessary to define the sites are relative to the body height of the person. This highly accurate and reliable US approach avoids compression artefacts, distinguishes between fat tissue and embedded structures, is not invasive, does not use ionising radiation, and is easily applicable in the field.

However, it cannot be assumed that the mean thickness value of these eight standardised sites that were selected for fat patterning analyses is the best representation of the real mean SAT thickness which is needed to calculate the fat mass. Therefore, extended measurement series containing many more (and randomly selected) sites are necessary to calibrate the mean obtained from the eight standardised sites^16,17^.

## Results

**Figure 1:**
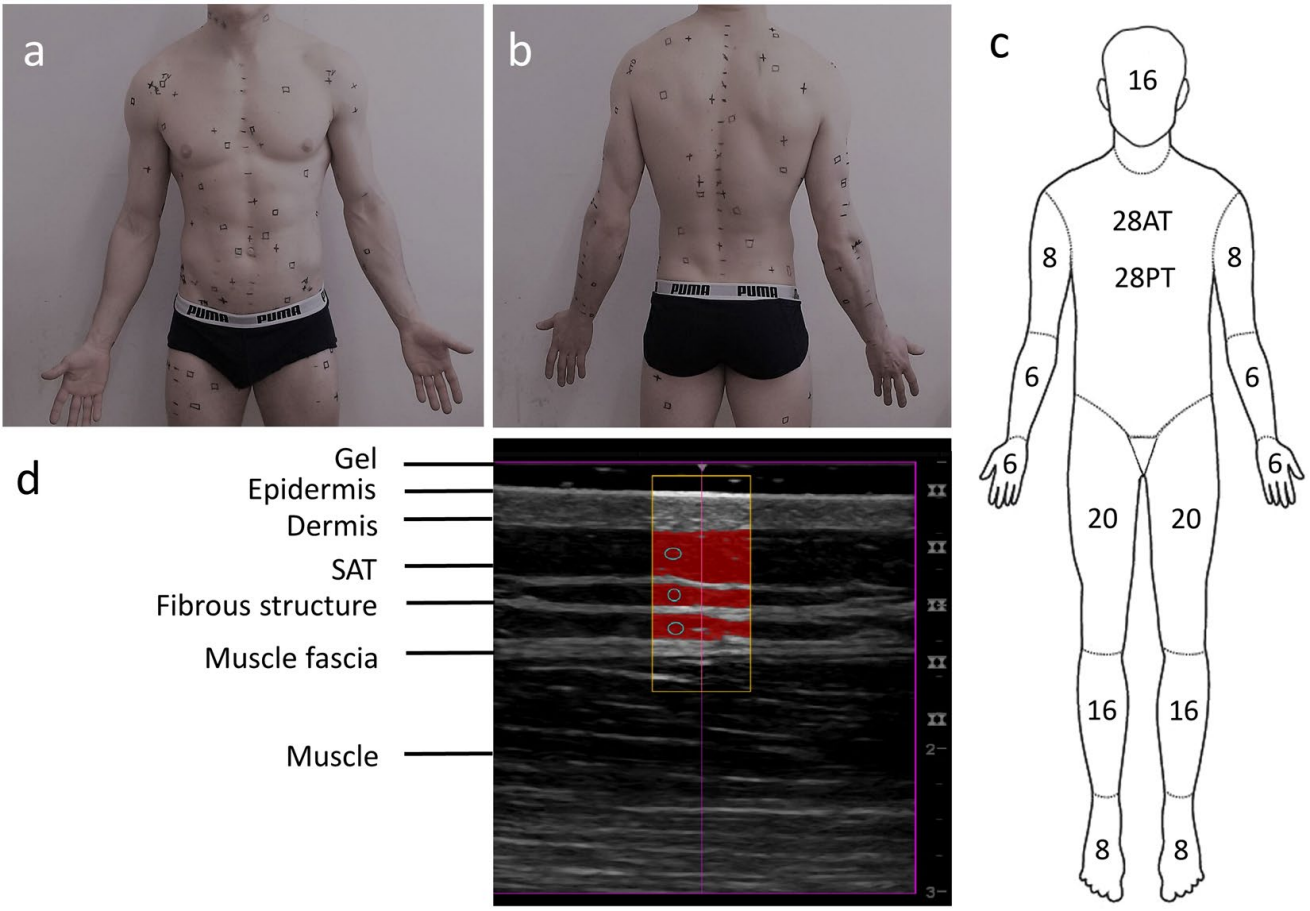
Ultrasound (US) measurement of mean subcutaneous adipose tissue (SAT). (**a,b**) exemplarily show randomly distributed measurement sites on the upper body of one of the ten participants. Crosses (+) belong to the first measurement series of 108 sites distributed all over the body, squares (□) to the second series. (**c**) Schematic drawing according to Lund and Browder^32^ indicating the 12 body parts. The segment genitalia was ignored in our study. In order to obtain integer numbers also for subsets of 108 and 54 sites, the following numbers of sites on the individual body parts were used: head HE (7%, 16 sites), neck NE (2%, 4 sites), anterior trunk AT (13%, 28 sites), posterior trunk PT (13%, 28 sites), buttocks BU (5%, 12 sites), upper arms AU (8%, 16 sites), forearms FA (6%, 12 sites), hands HA (5%, 12 sites), thighs TH (19%, 40 sites), legs LE (14%, 32 sites), and feet FE (7%, 16 sites). (**d**) Example of an evaluated US image. The red area represents the SAT in the region of interest (ROI). Marked are: the thick layer of US gel (which prevents compression), the epidermis, dermis, SAT, embedded fibrous structure, the fascia of the muscle, and the muscle underneath. In this example, the semi-automatic image evaluation software (USTissue Scientific - FAT Analysis Tool) measured 92 thicknesses with a mean value of *d*_I_ = 6.95 mm, and *d*_E_ = 5.51 mm. The *d*_I_ value includes the thickness of the fibrous structures, *d*_E_ represents the SAT without fibrous structures.

In a group of ten male participants (Table 1), subcutaneous adipose tissue (SAT) was measured twice at eight standardised sites using a recently developed ultrasound (US) method^16,17^. As an example, the US image of SAT at one of the eight standardised sites (lateral thigh, LT) is shown in Fig. 1d. In addition, SAT was also measured with the same US technique at 216 sites that were randomly distributed all over the body (Fig. 1a–c). The sums of the eight SAT thicknesses (*D*) of all 10 participants are shown in Table 2, and mean values of these eight measurements (d_M8_) are presented in Table 3. Thicknesses including the fibrous structures embedded in the SAT are indicated by the index “I”, measurements where these structures were excluded are indicated by the index “E”, and “F” indicates the thicknesses of the fibrous structures. For SAT thicknesses at individual measurement sites, the lower case letter “*d*” is used, and for the sums obtained from the eight sites at each participant, capital “*D*” is used. Table 2 also presents the surface areas (*S*) of the participants according to DuBois^29^, Haycock^30^, and Mosteller^31^, and also the means (*S*_M_) of these three.

**Table 1:** Characteristics and anthropometric data of participants.

	Unit	P1	P2	P3	P4	P5	P6	P7	P8	P9	P10	M	SD	MD	MAX	MIN
A	y	21	26	27	23	22	26	20	21	21	31	23.8	3.6	22.5	31	20
*m*	kg	66.0	64.6	71.6	95.1	72.4	62.5	96.2	85.6	84.1	92.7	79.1	13.2	78.3	96.2	62.5
*h*	m	1.816	1.749	1.813	1.919	1.674	1.751	1.840	1.941	1.903	1.853	1.826	0.084	1.828	1.941	1.674
*s*	m	0.940	0.947	0.964	1.060	0.892	0.911	1.065	1.014	0.982	1.017	0.979	0.059	0.973	1.065	0.892
*w*	m	0.831	0.716	0.745	0.773	0.735	0.776	0.883	0.830	0.728	0.693	0.771	0.060	0.759	0.883	0.693
*g*	m	0.980	0.898	0.924	0.936	0.910	0.941	1.044	1.055	0.899	0.887	0.947	0.060	0.93	1.055	0.887
*b*	m	0.337	0.298	0.300	0.345	0.300	0.325	0.367	0.363	0.329	0.334	0.330	0.025	0.332	0.367	0.298
*t*	m	0.524	0.469	0.464	0.499	0.468	0.510	0.602	0.526	0.494	0.483	0.504	0.041	0.497	0.602	0.464
BMI	kgm^−2^	20.0	21.1	21.8	25.8	25.8	20.4	28.4	22.7	23.2	27.0	23.6	2.9	23.0	28.4	20.0
MI_1_	kgm^−2^	20.5	20.7	21.7	24.8	25.7	20.8	26.0	23.1	23.9	26.1	23.3	2.3	23.5	26.1	20.5
C	1	0.516	0.526	0.518	0.513	0.523	0.532	0.549	0.522	0.541	0.531	0.527	0.011	0.524	0.549	0.513
W	1	0.44	0.40	0.41	0.45	0.41	0.43	0.48	0.43	0.42	0.38	0.42	0.03	0.42	0.48	0.38

The group included young normal or slightly overweight males (BMI_min_ = 20.0 kgm^−2^; BMI_max_ = 28.4 kgm^−2^). The table shows the individual values, mean values (M), standard deviations (SD), median (MD), maximum (MAX), and minimum (MIN) values of the following personal data: A (age), m (body mass), *h* (body height), *s* (sitting height), *w* (waist girth), *g* (gluteal girth), *b* (biceps girth flexed and tensed), *t* (thigh girth). Additionally, the following indices are included: body mass index (BMI), mass index (MI_1_), cormic index (C), and the waist to height ratio (W).

**Table 2:** Sums of subcutaneous adipose tissue from eight sites.

	Unit	P1	P2	P3	P4	P5	P6	P7	P8	P9	P10	M	SD	MD	MAX	MIN
*D*_I_	mm	23.5	28.4	31.8	36.0	39.7	45.5	51.6	59.6	62.1	81.6	46.0	18.0	42.6	81.6	23.5
*D*_E_	mm	18.5	23.2	25.9	30.9	34.9	39.2	44.9	53.5	51.8	74.6	39.8	17.0	37.1	74.6	18.5
*D*_F_	mm	5.0	5.2	5.9	5.1	4.8	6.3	6.7	6.1	10.3	7.0	6.2	1.6	6.0	10.3	4.8
*D*_F_/*D*_I_	1	0.21	0.18	0.19	0.14	0.12	0.14	0.13	0.10	0.17	0.09	0.14	0.04	0.14	0.21	0.09
S_1_	m²	1.85	1.79	1.91	2.25	1.82	1.76	2.19	2.17	2.12	2.17	2.0	0.19	2.02	2.25	1.76
S_2_	m²	1.82	1.77	1.90	2.26	1.85	1.74	2.24	2.14	2.11	2.20	2.0	0.21	2.00	2.26	1.74
S_3_	m²	1.82	1.77	1.90	2.25	1.83	1.74	2.22	2.15	2.11	2.18	2.0	0.20	2.00	2.25	1.74
S_M_	m²	1.83	1.78	1.90	2.25	1.83	1.75	2.22	2.15	2.11	2.18	2.0	0.20	2.01	2.25	1.75
VSAT_I_	dm³	3.49	4.05	5.23	6.73	6.16	6.45	10.70	9.45	9.75	13.42	7.54	3.18	6.59	13.42	3.49
VSAT_E_	dm³	2.82	3.26	4.45	5.63	5.05	5.49	9.61	8.31	8.16	12.09	6.49	2.96	5.56	12.09	2.82
TSAT_I_	kg	3.21	3.73	4.81	6.19	5.67	5.94	9.84	8.69	8.97	12.35	6.94	2.92	6.06	12.35	3.21
TSAT_E_	kg	2.59	3.00	4.09	5.18	4.65	5.05	8.84	7.64	7.51	11.12	5.97	2.73	5.12	11.12	2.59
TSAT_I%_	%	4.9	5.8	6.7	6.5	7.8	9.5	10.2	10.2	10.7	13.3	8.56	2.64	8.66	13.32	4.86
TSAT_E%_	%	3.9	4.6	5.7	5.4	6.4	8.1	9.2	8.9	8.9	12.0	7.33	2.51	7.25	12.	3.93

The table presents the sums (*D*) of subcutaneous adipose tissue (SAT) thicknesses measured at the eight standardised sites: *D*_I_ (fibrous structures included), *D*_E_ (fibrous structures excluded), *D*_F_ (fibrous structures). S_1,_ S_2_, and S_3_ represent the surface areas according to DuBois^29^, Haycock^30^, and Mosteller^31^, respectively. The total SAT volume VSAT [dm³] = *d*_M216_ [mm] ∙ S_M_ [m²]. The total SAT mass in kg: TSAT = VSAT ∙ ρ, with ρ = 0.92 [kg dm^−3^] for the density of fat^33^. TSAT[%] = 100 ∙ TSAT/m.

**Table 3:** Measurement of mean SAT.

	P1	P2	P3	P4	P5	P6	P7	P8	P9	P10	M
*d*_IM8_	2.94	3.55	3.98	4.50	4.97	5.69	6.45	7.46	7.76	10.20	5.75
*d*_IM216_	1.91	2.28	2.75	2.98	3.36	3.69	4.83	4.39	4.62	6.14	3.69
*d*_IM108a_	1.99	2.18	2.72	2.88	3.58	3.91	4.26	4.53	4.44	6.14	3.66
*d*_IM108b_	1.82	2.39	2.77	3.09	3.14	3.47	5.40	4.24	4.79	6.15	3.73
*d*_EM8_	2.32	2.90	3.24	3.87	4.36	4.90	5.61	6.69	6.48	9.33	4.97
*d*_EM216_	1.54	1.84	2.34	2.50	2.76	3.14	4.34	3.86	3.86	5.54	3.17
*d*_EM108a_	1.60	1.74	2.32	2.41	2.95	3.34	3.75	4.00	3.69	5.56	3.14
*d*_EM108b_	1.48	1.93	2.35	2.58	2.56	2.95	4.93	3.72	4.04	5.51	3.20
*d*_FM8_	0.62	0.65	0.74	0.64	0.60	0.79	0.84	0.76	1.28	0.87	0.78
*d*_FM216_	0.37	0.44	0.41	0.49	0.60	0.55	0.49	0.53	0.75	0.61	0.52
*d*_FM108a_	0.39	0.44	0.40	0.46	0.63	0.57	0.51	0.54	0.75	0.58	0.53
*d*_FM108b_	0.34	0.45	0.42	0.51	0.58	0.53	0.47	0.52	0.75	0.64	0.52

Mean SAT thicknesses *d*_M_.

The upper part shows mean SAT thicknesses obtained from eight standardised sites (*d*_M8_)^16^, and from 216 randomised sites on the body of each of the ten participants. The latter measurements were performed in two series of 108 measurements each (*d*_M108_). The index I stands for fibrous structures **i**ncluded in the thickness measurement, E for **e**xcluded, and F for the thickness of the **f**ibrous structures.

**Figure 2:**
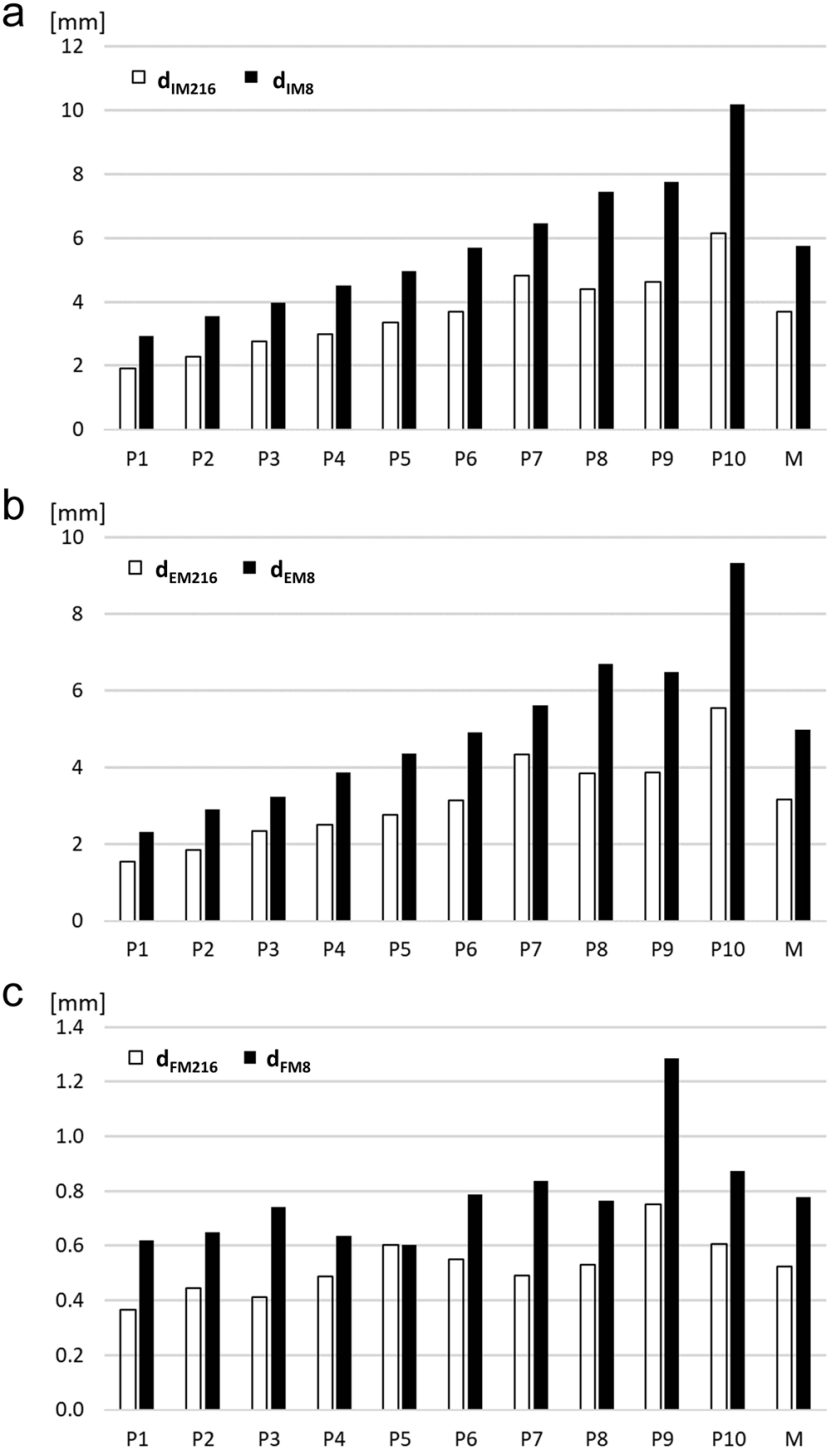
Mean SAT thicknesses. Black columns are the means (*d*_M8_) obtained from the measurements at the eight standardised sites. White columns are the means obtained from 216 sites (*d*_M216_) of each of the ten male participants. The participants P1 to P10 are ordered according to increasing means of *d*_IM8_. The columns labelled *d*_IM216_ and *d*_EM216_ represent the mean values of the ten participants. (i.e. the mean of all 2160 US measurements, each of them is a mean of typically 50 to 200 thickness measurements in each of the 2160 US images). (**a**) Mean thickness values including the embedded fibrous structures (*d*_IM_). (**b**) Mean thickness values excluding the embedded fibrous structures (*d*_EM_). (**c**) Mean thicknesses of fibrous structures (*d*_FM_ = *d*_IM_ − *d*_EM_)

The measurements at the 216 sites resulted in the reference means of SAT thicknesses for each of the participants (Table 3). A comparison of the SAT means obtained with the eight standardised sites^16,17^ is presented in Fig. 2. Means of typically 50 to 300 measurements obtained from each US image were used to represent the SAT thickness at a given individual site.

**Figure 3:**
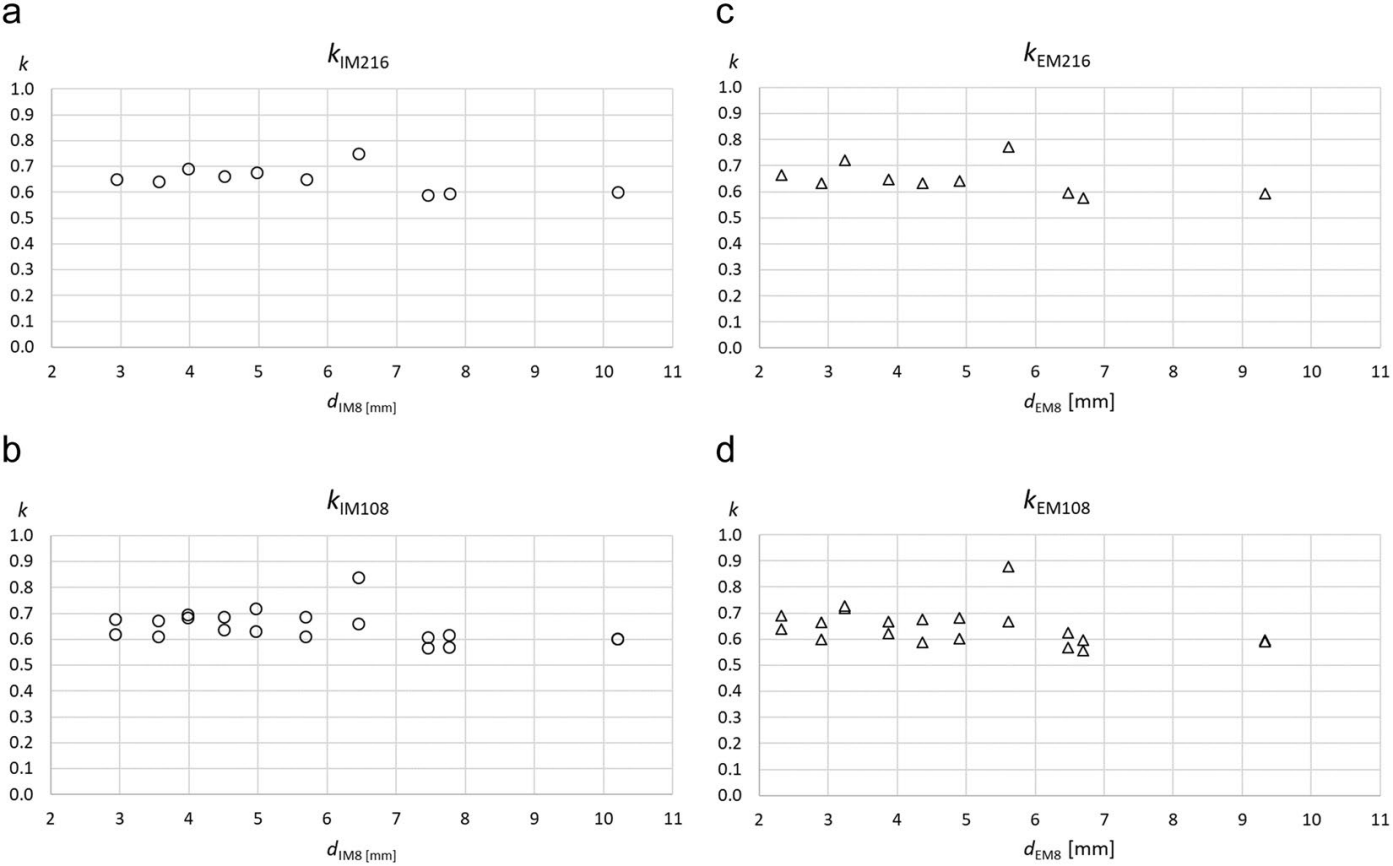
Mean SAT thicknesses at eight standardised sites compared to measurements at 216 and at 108 sites. Ten participants were measured at 216 sites (in two series of 108 sites each) distributed randomly all over the body (compare to Fig. 1a–c), and also at the eight standardised sites. The ten participants are ordered according to increasing mean SAT thickness (*d*_IM8_). The mean SAT thicknesses (*d*_M_) obtained from measurements at 216 (or at 108) sites deviate by a factor *k* from the means of the eight standardised sites: *k*_M216_ = *d*_M216_/*d*_M8_, and analogously *k*_M108_ = *d*_M108_/*d*_M8_. The factors for SAT thicknesses **i**ncluding fibrous structures (I) are shown in (**a**), and the comparisons with the two subgroups of 108 sites each are shown in (**b**). (**c,d**) display the factors for thicknesses with fibrous structures **e**xcluded (E).

The mean thicknesses obtained from the eight standardised sites deviated from the means obtained from the 216 randomised sites. The factor *k* represents this for the individual ten participants (Table 4). For measurements that included (index “I”) fibrous structures the calibration equation is: *d*_IM216_ = *d*_IM8_ ∙ *k*_IM216_, and for measurements that exclude (index “E”) fibrous structures: *d*_EM216_ = *d*_EM8_ ∙ *k*_EM216_. The table also shows the factors when the eight-site measurements are compared to the 108-site measurements (the measurement series of 216 sites was taken in two series of 108 sites each). The k-values corresponding to 216 measurements with fibrous structures included (I), and without (E), are shown in Fig. 3a,c, and for the 108-site measurement series in Fig. 3b,d. Mean k values were 0.65 in both cases.

**Table 4:** Individual mean SAT thickness calibration factors *k*_M _for the ten participants.

	P1	P2	P3	P4	P5	P6	P7	P8	P9	P10
*k*_IM216_	0.65	0.64	0.69	0.66	0.68	0.65	0.75	0.59	0.59	0.60
*k*_IM108a_	0.68	0.61	0.68	0.64	0.72	0.69	0.66	0.61	0.57	0.60
*k*_IM108b_	0.62	0.67	0.70	0.69	0.63	0.61	0.84	0.57	0.62	0.60
*k*_EM216_	0.66	0.63	0.72	0.65	0.63	0.64	0.77	0.58	0.60	0.59
*k*_EM108a_	0.69	0.60	0.72	0.62	0.68	0.68	0.67	0.60	0.57	0.60
*k*_EM108b_	0.64	0.67	0.73	0.67	0.59	0.60	0.88	0.56	0.62	0.59
*k*_FM216_	0.59	0.69	0.55	0.77	1.00	0.70	0.59	0.69	0.59	0.70
*k*_FM108a_	0.63	0.67	0.54	0.73	1.04	0.73	0.61	0.71	0.58	0.66
*k*_FM108b_	0.56	0.70	0.57	0.80	0.97	0.67	0.56	0.68	0.59	0.73

The calibration factor *k*_M216_ is defined as: *k*_M216_ = *d*_M216_/*d*_M8_, and analogously *k*_M108_ = *d*_M108_/*d*_M8_. The idex I indicates that fibrous structures are **i**ncluded; E stands for **e**xcluded.

**Figure 4:**
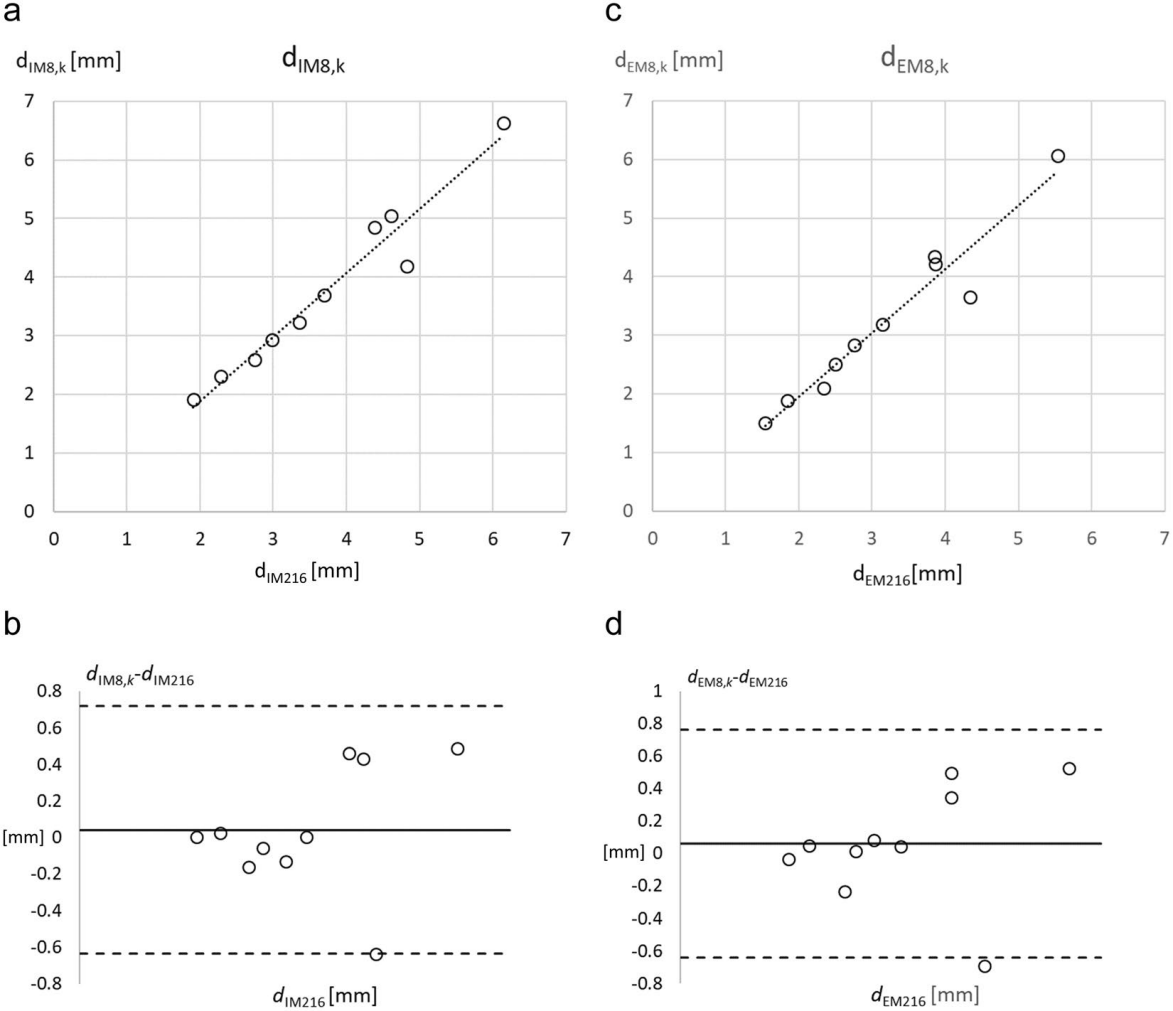
Application of the calibration factor k. The application of the mean calibration factor *k* = 0.65 is used to calibrate the mean SAT thicknesses obtained in the ten participants from the eight standardised sites: *d*_M8,k_ = 0.65 *d*_M216_. In (**a** and **c**) *d*_M8,*k*_ is displayed over *d*_M216_. Pearson’s correlation coefficient was used. Data was normally distributed. (**a**) Embedded fibrous structures included (I): R² = 0.951, SEE = 0.344 mm. (**c**) Embedded fibrous structures excluded (E): R² = 0.938, SEE = 0.363 mm. (**b**) presents Bland-Altmann plots for I: mean M = 0.04 mm, standard deviation SD = 0.35 mm, limits of agreement are [−0.63, 0.72] mm; for E (**d**): 0.06 mm, 0.36 mm, [−0.64, 0.76] mm.

Figure 4a and c show the correlations between the mean thicknesses obtained for the 216 randomised sites (*d*_M216_) and the calibrated means (*d*_M8,*k*_) from the eight standardised sites according to: *d*_M8,*k*_ = *k* ∙ *d*_M8_. The correlation coefficient R² was 0.95 (p < 0.01), and SEE was 0.34 mm for data including fibrous structures (Fig. 4a), and R² was 0.94 (p < 0.01), and SEE 0.36 mm without fibrous structures (Fig. 4c). Figure 4b and d show the respective limits of agreement [−0.63, 0.72] mm, and [−0.64, 0.76] mm, respectively.

**Figure 5:**
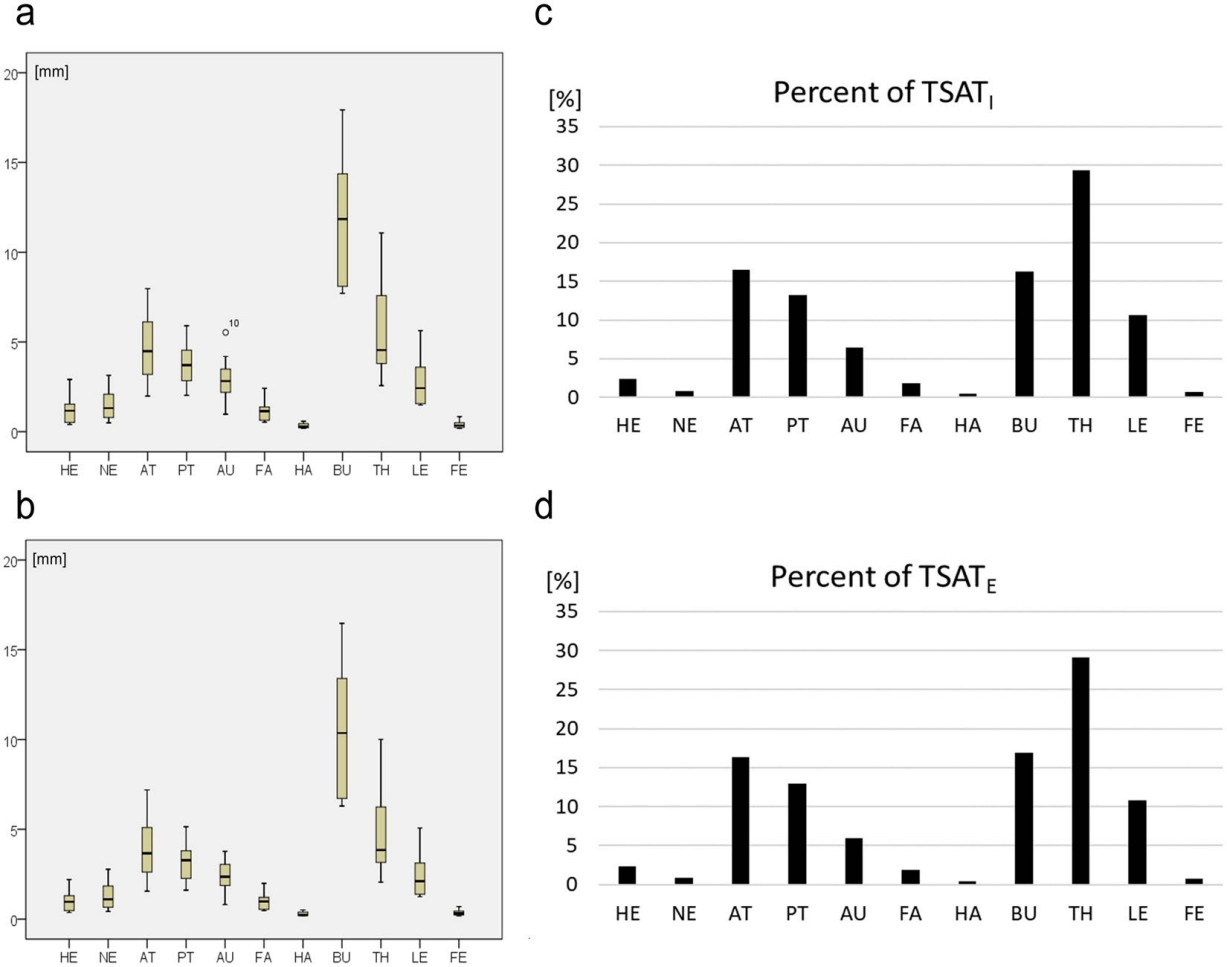
Mean SAT thicknesses (*d*_M,BS_) at the 11 body segments (BS). This data set resulted from 10 ∙ 216 = 2160 US images, captured at 11 body segments (for abbreviations see Fig. 1). The individual values of the ten male participants are shown in Table 5. (**a**) Mean SAT thickness of each segment (compare to Fig. 1c) with fibrous structures included (*d*_IM,BS_). (**b**) Data for thicknesses where fibrous structures were excluded (*d*_EM,BS_). (**c,d**) The columns show the percentages (SAT_%,BS_) of the body segment contributions to the total subcutaneous adipose tissue (TSAT). The mean SAT thicknesses M_I,2160_ and M_E,2160_ (mean of 2160 SAT thickness measurements) of all 10 participants measured at 216 sites each were 3.69 mm and 3.17 mm, respectively (see Table 3); therefore, normalisation factors of *f*_I_ = 100/3.6947 = 27.07, and *f*_E_ = 100/3.1704 = 31.54 result. For SAT thicknesses with fibrous structures included (I) we get SAT_I,%,BS_ = *d*_IM,BS_ ∙ *f*_I_ ∙ S_BS_/S, and with fibrous structures excluded (E) SAT_E,%,BS_ = *d*_EM,BS_ ∙ *f*_E_ ∙ S_BS_/S.

Mean SAT thicknesses of the 11 body segments (BS) head, neck, anterior trunk, posterior trunk, upper arms, forearms, hands, buttocks, thighs, legs, an feet (*d*_M,BS_) are presented in Fig. 5a,b and Table 5. Highest mean value was 12 mm at buttocks and lowest was 0.3 mm at hands. The SAT percentages (SAT_%,BS_) for each segment are presented in Fig. 5c,d and Table 5. The columns represent the percentages of the SAT volumes (and thus also of the fat mass percentages) of the 11 body segments.

**Table 5:** Mean SAT thicknesses (*d*_M,BS_) and SAT percentages of the 11 body segments (BS). The indices I and E stand for fibrous structures **i**ncluded in the thickness measurement and E for **e**xcluded. SAT thicknesses of the ten participants were measured at 216 sites each (in two series of 108 each). The numbers of sites on each of the 11 body segments according to Lund and Browder^32^ were chosen proportionally to the surface areas of these segments (Fig. 1c): 16 on head (HE), 4 on neck (NE), 28 on anterior trunk (AT), 28 on posterior trunk (PT), 16 on upper arms (AU), 12 on forearms (FO), 12 on hands (HA), 12 on buttocks (BU), 40 on thighs (TH), 32 on legs (LE), and 16 on feet (FE). The according box plots are shown in Fig. 5(a,b). The mean percentages (of all ten participants) of the fat mass contributions of the individual body segments are also shown (SAT%,BS); this sums up to 99% (genitalia, wich represent 1%, were ignored).

	P1	P2	P3	P4	P5	P6	P7	P8	P9	P10	MD	M	SAT_%,BS_
*d*_IM,HE_	0.52	1.08	1.27	0.49	2.20	2.91	1.25	0.40	1.06	1.54	1.17	1.27	2.4
*d*_IM,NE_	0.50	1.20	1.51	1.19	2.09	2.45	0.67	0.79	3.14	1.43	1.31	1.50	0.8
*d*_IM,AT_	1.98	3.19	4.13	2.90	4.14	4.84	5.27	6.40	6.12	7.98	4.49	4.69	16.5
*d*_IM,PT_	2.66	2.03	3.56	3.20	2.84	4.04	3.86	4.54	4.99	5.91	3.71	3.76	13.2
*d*_IM,AU_	0.97	2.19	2.02	2.74	3.29	2.65	3.49	2.90	4.19	5.53	2.82	3.00	6.5
*d*_IM,FA_	0.52	0.58	1.37	0.63	0.99	1.30	1.61	0.73	2.41	1.37	1.15	1.15	1.9
*d*_IM,HA_	0.22	0.26	0.19	0.36	0.45	0.58	0.55	0.20	0.28	0.22	0.27	0.33	0.4
*d*_IM,BU_	7.71	8.10	7.92	12.62	10.83	12.85	16.99	17.93	11.08	14.36	11.85	12.04	16.3
*d*_IM,TH_	2.57	3.28	3.80	4.58	4.25	4.52	8.49	7.06	7.59	11.08	4.55	5.72	29.4
*d*_IM,LE_	1.53	1.48	1.56	2.03	3.23	2.64	4.08	2.22	3.60	5.63	2.43	2.80	10.6
*d*_IM,FE_	0.19	0.24	0.24	0.37	0.84	0.50	0.56	0.33	0.35	0.31	0.34	0.39	0.7
*d*_EM,HE_	0.48	0.87	1.14	0.43	1.73	2.19	1.06	0.37	0.76	1.30	0.97	1.03	2.3
*d*_EM,NE_	0.43	0.99	1.35	1.02	1.84	2.16	0.62	0.67	2.77	1.19	1.10	1.30	0.8
*d*_EM,AT_	1.55	2.61	3.59	2.35	3.51	3.73	4.53	5.75	5.09	7.20	3.66	3.99	16.4
*d*_EM,PT_	2.00	1.60	3.12	2.64	2.26	3.60	3.43	3.80	4.10	5.14	3.27	3.17	13.0
*d*_EM,AU_	0.81	0.96	1.86	2.29	2.42	2.13	3.05	2.98	3.50	3.77	2.35	2.38	6.0
*d*_EM,FA_	0.50	0.47	1.21	0.55	0.86	1.11	1.43	0.57	1.98	1.19	0.99	0.99	1.9
*d*_EM,HA_	0.22	0.22	0.19	0.29	0.40	0.47	0.51	0.20	0.24	0.21	0.23	0.29	0.5
*d*_EM,BU_	6.49	6.72	6.30	11.04	9.42	11.43	16.25	16.47	9.70	13.41	10.37	10.72	16.9
*d*_EM,TH_	2.05	2.53	3.15	3.73	3.35	3.95	7.50	6.15	6.24	10.01	3.84	4.87	29.2
*d*_EM,LE_	1.24	1.24	1.37	1.70	2.70	2.33	3.74	1.89	3.12	5.07	2.11	2.44	10.8
*d*_EM,FE_	0.18	0.23	0.22	0.36	0.70	0.45	0.52	0.26	0.34	0.30	0.32	0.36	0.8

## Discussion

A representative mean SAT value is important for correct calculation of the total subcutaneous adipose tissue (SAT) mass. For this purpose, the mean obtained from the eight sites needs to be calibrated by a representative mean obtained from a large number of sites distributed randomly all over the body. The classification of body segments according to Lund and Browder (Fig. 1c)^32^ implies to use: 4 sites on the head, 1 on neck, 7 on anterior trunk, 7 on posterior trunk, 3 on buttocks, 4 on upper arms, 3 on forearms, 3 on hands, 10 on thighs, 8 on legs, and 4 on feet. This would amount to 54 (the genital area was neglected). Several series of 54 sites in the same individual (pilot study, not shown) indicated that scattering of the means of the 54 sites was too high for the purpose of this calibration study. When using 108 sites, scattering is still noticeable as can be seen in Fig. 3b,d. Therefore, we used 216 sites corresponding to about one site per dm^2^ in adults.

The mean values of the individual calibration factors (Table 4) were 0.65 for both *k*_I__M216_ and *k*_EM216_ (derived from comparisons of 2160 measurements at randomly chosen sites - 216 measurements in ten participants - and additional two times eight measurements at the standardised sites; this amounts to 2320 US measurements). Each thickness measurement at an individual site was represented by the mean of typically 100 thickness values (depending on the breath of ROI setting) measured by the evaluation software (amounting to more than 200,000 thickness values). Although there is still a rest of scattering when using 216 sites due to the randomisation, the standard deviation of the mean calibration factor is low because it is obtained from all ten persons which reduces the “randomisation”-scattering. The means obtained from the eight sites for each individual participant in this group of male participants ranged from 2.9 to 10.2 mm, and the corresponding means obtained from the 216 randomised sites ranged from 1.9 to 6.1 mm.

The mean of eight sites used in the standardised ultrasound method for studying SAT patterning^16,17^ overestimated the mean obtained from 216 randomly distributed sites in all individual cases (Fig. 2a,b, Table 3). This overestimation is not surprising as the standardised eight sites were developed to investigate the fat patterning of the body and therefore includes some of the main fat depot areas for subcutaneous fat deposition (femero-gluteal region, back, and anterior abdominal wall). These fat depot areas are represented by five of the eight sites (FT, LT, ES, UA, and LA). The mean calibration factor *k* = 0.65 (derived from values in Table 4; the SD of *k* was 0.06) is used here to correct this overestimation for both thickness measurements with fibrous structures included (*d*_IM8,*k*_ = 0.65 ∙ *d*_IM8_) and excluded (*d*_EM8,*k*_ = 0.65 ∙ *d*_EM8_). For the measurement series at eight sites (*d*_IM8,*k*_) compared to 216 sites (*d*_IM216_), R^2^ was 0.95 (p < 0.01), the SEE was 0.34 mm, and LOA were [−0.63, 0.72] mm. This scattering transforms linearly when the calibrated mean SAT thickness *d*_IM8,*k*_ (or *d*_EM8,*k*_ for the assessment of SAT without fibrous structures0 is used for calculating total SAT volume according to: volume is mean thickness times surface area S (S can be measured accurately by a calibrated scanning system, or determined approximately according to surface area formulas^29–31^). For SAT mass determination, we used the density of ρ = 0.92 for fat^33^.

### Limitations and further developments

1. Detailed SAT and fat density values of humans as functions of site, temperature, age, and hydration status are missing. Ethics permission for such studies of human SAT in our lab is given already, and these measurements will start in due course. All imaging methods that can be used for fat mass determination based on volumetry will benefit from accurately determined SAT density in humans.

2. Body surface was determined only approximately in this study using DuBois’ data and related formulas and not with the high accuracy obtainable with a state-of-the-art 3D scanner because the focus of this study is on minimising the error due to SAT thickness means obtained by US from only eight (standardised) sites.

3. Determination of SAT mass (or pure subcutaneous fat) mass by US as used here does not capture visceral fat. Attempts to assess visceral fat by US have been made by other groups^37,34^, however, US only detects surrogate parameters like intra-abdominal distances. MRI is capable of measuring visceral fat, although fat layer thickness measurements do not reach the accuracy of US thickness measurements because the pixel size is typically 1.3 to 2 mm in total body scans, and MRI measurement sequence and image segmentation protocols are not standardised for this purpose. Development of improved MRI methods towards higher standards for fat studies is in progress in our laboratory. Comparisons of SAT (measured by US) with VAT (measured by MRI) will show the possibilities and limitations of total body fat (TBF) assessment (on the anatomical level) based on US SAT measurements solely. We assume that there is a good chance to find useful correlations for acceptable assessment accuracy because SAT, which can be determined with high accuracy by US, accounts for typically about 80% of TBF^35–37^ and therefore scattering of the VAT percentage can be expected to have minor effect on the TBF assessment error. However, there may be outliers, particularly in groups with obesity or extreme underweight. Meanwhile, we use the waist to height ratio (W = *w*/*h*), which is the most important anthropometric predictor of health and premature death caused by obesity^38^, as a surrogate measure for VAT. The studies in progress will also show whether a combined approach (US SAT measurements and anthropometric indices like W) will improve the assessment accuracy.

4. In our group of ten male participants, BMI ranged from 20.0 to 28.4 (Table 1), and total SAT percentage from TSAT_I%_ = 4.9% to 13.3% (mean was 8.6% of body mass; Table 2), which represents a group ranging from normal weight to overweight (according to the WHO definitions)^39^. In groups with underweight, SAT thickness of fat depots may get closer to the SAT thickness of other sites; preliminary studies indicate that a higher calibration factor can be expected in such cases. The investigation presented here includes young white Caucasian males. Therefore, similar studies with females, and with other ethnic groups will be of interest, although it would be surprising when results deviated substantially in such cases because the eight sites cover a representative set of fat depot sites, and differences from site to site in different groups can be expected to equal out to a large extend when means of all eight sites are taken. A future focus should also be on older adults and on children, who are not included here.

5. Eight representative sites were chosen because the development of the US method started out from the eight sites that have been used since many years by ISAK (International Society for the Advancement of Kinanthropometry) for skinfold measurements^14,15,28^. It turned out that several of these ISAK sites are not well suited for US measurements (because of complex underlying anatomical structures that are difficult to interpret), and that marking of these sites is difficult and time consuming. Therefore, most of them had to be replaced by new ones; however, the number of eight sites remained. Data mining studies will show whether this number or possibly a reduced number of sites will be the optimum choice for SAT (and for TBF) mass assessment based on US.

## Methods

### Participants

50 young male participants with BMI values below 28.5 kgm^−2^ were investigated at the eight standardised US sites and ten were selected out of this set to cover a range of mean SAT thicknesses (from eight sites) from approximately 3 mm to 10 mm (with fibrous structures included: *d*_IM8_). According to a preliminary schedule and discussion basis^40^ for defining SAT ranges, this group represents the SAT categories “desirable range” (2.5 to 7.5 mm) and “noticeable ballast weight” (*d*_IM8_ from 7.5 to 12.5 mm) for male persons of the general public. For competitive male athletes, according to the above-mentioned preliminary schedule, the desirable range is 2.5 to 3.8 mm, noticeable ballast weight 3.8 to 6.3 mm, and considerable ballast weight is above 6.3 mm). For anthropometric data see Table 1. Permission for the study was provided by the ethics committee of the Medical University of Graz (20–295 ex08/09).

### Informed consent

All participants received an information letter and completed a written consent form.

### Standardised sites for US measurements

The standardised eight sites described by Müller *et al*.^16^, and Störchle *et al*.^17^ were used: upper abdomen (UA), lower abdomen (LA), erector spinae (ES), distal triceps (DT), brachioradialis (BR), lateral thigh (LT), front thigh (FT) and medial calf (MC). Two measurement series were performed and the mean was used. Sites were marked on the right side of the body in a standing (UA, LA, LT) or sitting (ES) position or with the arm (DT, BR), or the leg (FT and MC) supported. All US measurements at these eight sites were made with the participants lying in a supine (UA, LA, BR, FT), prone (ES; DT) or rotated position (LT, MC)^16,17^.

### Series of 216 SAT measurements in each of the ten participants (two series of 108 measurements each)

The 216 sites were randomly distributed all over the body (Fig. 1a–c). Eleven body segments (Fig. 1c) were covered with a number of sites proportional to their contribution to the total surface area. The body segments were chosen in accordance with the criteria of Lund and Browder^32^, but without the segment “genitalia” (which was ignored here). The surface areas of the head, hand, and foot correspond to 7%, 5%, and 7% of the total surface area and should therefore be represented by 16, 12, and 16 measurements, respectively. However, it is extremely difficult to find such a high number of useful sites on hands and feet because of many vessels and complex anatomical structures there. Therefore, only half of the corresponding site numbers were measured and these values were considered twice. As it is very inconvenient for the participant to measure 16 sites on the head, therefore the same approach was used for the head too.

### Ultrasound imaging of SAT

US imaging is based on the pulse-echo technique. A series of US pulses (each several wavelengths long) is sent into a given tissue. Medical diagnostic US systems conventionally use c = 1540 ms^-1^ for distance (*d*_US_) determination in soft tissue. In adipose tissue, the speed of sound is lower: 1450 ms^-1^^ 16,33,41^, and therefore, this appropriate speed of sound is used for thickness measurements in this study (where mean SAT thicknesses ranged from 1.9 to 6.1 mm). Goss *et al*.^42^ list speed of sound measurements (performed in 1953) in human fat: for SAT in which connective tissue was removed, 1459 ms^-1^ were measured, i.e. a value that does not deviate noticeably from the value of 1450 ms^-1^ used here^33^: For a 6 mm thick fat layer, a difference of 10 ms^-1^ would result in a distance measurement error of only 0.04 mm, which is far below the measurement error due to the limited US resolution. In thick layers, the correct choice of sound speed can noticeably influence the measurement error^17^, however, for example in a fat layer as thick as 30 mm, the error due to a sound speed difference of 10 ms^-1^ is only 0.2 mm, which is still within the range of the error determined by the image resolution. In this US method, the thicknesses *d*_E_ measure SAT without embedded fibrous structures, whereas *d*_I_-values include these connective tissues. Values of the speed of sound in connective tissue embedded in SAT are not available, but even a speed of sound of 1500 ms^-1^ (for comparison, in the arterial wall tissue 1501 ms^-1^ were measured^42^) would not noticeably effect measurement accuracy: assuming 50 ms^-1^ sound speed deviation (from the used 1450 ms^-1^), about 0.2 mm distance difference would result in a 6 mm thick SAT layer (in human SAT, typically about 15% is connective tissue, and this percentage decreases with increasing SAT thickness). Such possible measurement errors cannot be corrected currently because the speed of sound in the particular connective tissue embedded in SAT is not known yet. Again, these possible errors are small compared to the reliability errors and when compared to the furrowed borders of SAT.

All US measurements, at both the standardised eight sites and at the randomised 216 sites, were performed according to the standardised approach^16,17^. All US images clearly showed the lower margin of the skin and the upper margin of the muscle fascia (Fig. 1d). To avoid compression of fat, the probe was placed above a given site by using a thick layer of US gel between the probe and the skin (typically about 5 mm). Measurements were carried out using a GE Logiq-e US device and the linear probe L8-18i operated at 9 to 18 MHz.

### Interactive multiple thickness measurement of SAT layers

A semi-automatic evaluation algorithm designed for multiple interactive evaluations of SAT layer thicknesses was used to evaluate the mean SAT layer thickness in a given US image (USTissue Scientific - FAT Analysis Tool, www.iasms.org, www.rotosport.at). The number of thickness measurements in each individual image was typically 50 to 300 (depending on the selected region of interest breadth). In the evaluation software, sound speed was set to 1450 ms^-1^ for distance calculation in adipose tissue^16,33^. According to the standardised protocol^16,17^, the tissue segmentation was controlled visually and, if necessary, parameters that determine the accepted segment inhomogeneity were set manually to optimise the SAT contour detection. The software enables the operator to distinguish between distances in which fibrous structures are included (index “I”) or excluded (index “E”). Figure 1d shows an example of an evaluated US image. The centre lines in the image correspond to the centre of the US probe which was held exactly above the marked site. The rectangular ROI was usually set symmetrically to the centre line. The contour detection algorithm starts out from the manually set circles (or ellipses) and measures multiple thicknesses automatically. Using a ROI that is set symmetrically to the centre line of the US image ensures that the thickness is determined at the marked site (the middle of the US probe is positioned at the marking on the skin, with a thick layer of gel in-between which avoids compression artefacts). A visual control makes sure that the algorithm detected the SAT layer correctly.

### Accuracy of US thickness measurements

Diffraction and technically obtainable minimum pulse length limit lateral and axial resolution approximately to the wavelength used. Diagnostic US probes (transducers) use frequencies from 3 to 22 MHz, which corresponds to a wavelength in soft tissue of 0.5–0.07 mm. US attenuation increases with increasing frequency—typical investigable depths are between 10 mm (22 MHz) and 200 mm (3 MHz). In this study, 18 MHz were used for thin layers with a border detection resolution of about 0.1 mm, and for thick fat layers, lower frequencies down to 9 MHz were used (resulting in a border detection resolution of about 0.2 mm). There is no relevant dependency of sound velocity on frequency for diagnostic US measurements of tissue thickness^43^. The calculated distance is: sound speed (in SAT) multiplied by half the echo time. The technical measurement error can be kept very low (about 0.2 mm at 18 MHz). The limiting accuracy factors are of biological nature because they are beyond the technically obtainable image resolution and measurement accuracy: furrowed tissue borders and viscous-elastic behaviours of SAT. The influence of these biological limitations is minimised because the image evaluation algorithm used takes mean values of many thickness measurements (typically about 100) in a given image which results in a standard error of the mean that is a magnitude lower than the standard deviation of the individual measurements^17^. A sound speed deviation from the “real” value of 10 ms^-1^ would result in 0.07 mm thickness difference when measuring a SAT layer of 10 mm, for example. Temperature of fat tissue underneath the skin (SAT) may range between about 30° and 35°. As the human body consist of about 70% of water, the speed of sound in soft tissue is largely determined by the speed of sound in water. For water, the temperature coefficient is well known^44^: it is about 0.14% per K. In other words: A temperature difference of 5° (and more is not to be expected when measurements of humans are made in room temperature, typical 23° to 25° in our laboratory) would result in 0.7%, i.e. a change from 30° to 35° would change the speed of sound from 1509 to 1520 m/s^44^. There are no data available that would give a reason to anticipate substantially larger effects in SAT. Detailed discussion of accuracy limitations have been published previously already^15–17^.

### Reliability of US measurements

To obtain maximum reliability, it is paramount to mark sites and capture images in accordance with the standardised US measurement technique^16,17^. In a recent publication, an inter-observer study was performed (three observers measured 12 athletes) where mean values of the eight standardised measurements (*d*_IM_) ranged from 1.25 to 6.25 mm; 95% of the mean values were within ±0.13 mm^16^. Results of intra-tester measurements performed in a group ranging from mean SAT thicknesses (*d*_IM_) of 1.6 to 30.6 mm (including normal weight, overweight, and obese persons) showed that 95% of the mean values (*d*_IM_) were within ±0.28 mm^17^.

### Statistics

For statistical analysis, SPSS (Version 24) was used. Normal distribution was tested by the Shapiro-Wilk test. Statistical analyses included the determination of standard errors of the estimate (SEE), linear regressions including coefficients of determination (R²), and significances (p < 0.01), and limit of agreement (LOA)^45^.

## Data Availability

All relevant data generated or analysed during this study are included in this article.
